# Hypertension with unilateral adrenal aldosterone and cortisol cosecreting adenoma: A case report

**DOI:** 10.1111/jch.14374

**Published:** 2021-10-17

**Authors:** Zhe Hu, Xin Chen, Yuan Shao, Fang‐Xiu Luo, Shao‐Li Chu, Ji‐Guang Wang

**Affiliations:** ^1^ Department of Hypertension Ruijin Hospital Shanghai Jiaotong University School of Medicine Shanghai China; ^2^ Department of Urology Ruijin Hospital Shanghai Jiaotong University School of Medicine Shanghai China; ^3^ Department of Pathology Ruijin Hospital Shanghai Jiaotong University School of Medicine Shanghai China

**Keywords:** primary aldosteronism, subclinical Cushing's syndrome, unilateral adrenal adenoma

## Abstract

Here, we report a case of unilateral adrenal aldosterone and cortisol co‐secreting adenoma. A 34‐year‐old man with a history of severe hypertension for one year was detected hypokalemia (2.42 mmol/L lowest) and unilateral adrenal mass in a size of 71 mm*63 mm. Measurements of plasma aldosterone concentration and plasma renin activity showed marked increases. Primary aldosteronism was diagnosed. To exclude adrenal malignancy, the function of zona fasciculate was evaluated, and 24‐h urine free cortisol was found abnormal in a testing. Further examinations revealed that circadian rhythm of serum cortisol disappeared and 2 mg‐dexamethasone suppression test was positive. The final diagnosis was secondary hypertension, primary aldosteronism and subclinical Cushing's syndrome. After unilateral adrenalectomy, his blood pressure was normalized and biochemical parameters in the normal range. In conclusion, in patients with a large aldosterone‐producing adenoma, the function of zona fasciculate might have to be evaluated for the identification of aldosterone and cortisol co‐secreting neoplasms.

## 1 INTRODUCTION

Primary aldosteronism with subclinical Cushing's syndrome used to be thought as a rare disease.^1^, ^2^ However, with the recent substantial increase in the diagnosis of primary aldosteronism, there was an increase in the reporting of cases with the coexistence of primary aldosteronism and subclinical Cushing's syndrome.^3^, ^4^, ^5^, ^6^, ^7^ Although subclinical Cushing's syndrome often has no clinical manifestations, its coexistence with primary aldosteronism is clinically relevant for the diagnosis and treatment of the latter disease. The lateralization of primary aldosteronism relies on the contrast of the aldosterone and cortisol concentrations. The hypersecretion of cortisol from the same adrenal gland may therefore influence the accuracy of lateralization. In addition, if the adrenal gland with both aldosterone and cortisol hypersecretion is surgically removed, the post‐operation management on cortisol may also be complicated, because the contralateral adrenal gland may have been suppressed in the secretion of cortisol. In this case, patients may need cortisol supplementation. The coexistence of primary aldosteronism and cortisolism therefore needs to be scrutinized in the diagnosis and treatment of primary aldosteronism. Here we report a case with unilateral adrenal aldosterone and cortisol cosecreting adenoma.

## 2 CASE SUMMARY

A 34‐year‐old man was admitted in the Department of Hypertension, Ruijin Hospital, Shanghai Jiaotong University School of Medicine, Shanghai, China for the evaluation of severe hypertension with hypokalemia and adrenal mass. Blood pressure elevation was found about 1 year ago, with symptoms of dizziness and fatigue. Systolic/diastolic blood pressure was not controlled (150‐160/110‐120 mmHg) with the use of irbesartan and amlodipine. In one of his clinic visits, hypokalemia was detected, the lowest serum potassium concentration was 2.42 mmol/L, and adrenal ultrasound examination showed right adrenal mass in a size of 71 mm*63 mm. He had no history of vomiting, diarrhea, drug abuse, chronic diseases other than hypertension, such as diabetes or hyperthyroidism, or surgery. He was married, and had two sons, all healthy.

His body mass index was 22.0 kg/m^2^, with a body weight of 55 kg and body height of 158 cm. Seated systolic/diastolic blood pressure was 160/112 mmHg. Physical examinations showed no central obesity, “moon face”, or purple or red striae, which could be seen from hypercortisolism. No positive signs were found on cardiopulmonary examinations, and no vascular bruit was heard in the abdomen and neck.

Laboratory tests revealed that fasting plasma glucose and serum lipids were in the normal range. Serum creatinine was normal. Both serum (2050 μg/L) and urinary (1729 μg/L) concentrations of β2 microglobulin were elevated. Urinary albumin‐to‐creatinine ratio was slightly elevated (3.73 mg/mmol), and 24‐h urinary protein excretion was 165 mg. Serum potassium was initially 2.71 mmol/L, and after potassium supplementation increased to 3.01 mmol/L, with a 24‐h urinary potassium excretion of 85 mmol. pH of urine was 8.0. Blood gas analysis showed metabolic alkalosis, with a pH value of 7.46.

Plasma aldosterone concentration and plasma renin activity (PRA) was measured by radioimmunoassay. Plasma aldosterone concentration was 339.6 and 715.7 pg/mL at the supine basal and standing excitation, respectively. The corresponding values were 0.01  and 0.04 ng/mL/h, respectively, for plasma renin activity and 33 960 and 17 893[> 300 (pg/mL)/(ng/mL/h^–1^)], respectively, for aldosterone‐to‐renin ratio. Twenty‐four hour urinary aldosterone excretion was 36.4 μg. Serum and 24‐h urine free cortisol (UFC), free triiodothyronine, free thyroxin, thyroid‐stimulating hormone, plasma metanephrine and normetanephrine, and urine catecholamines were all in the normal range.

**FIGURE 1:**
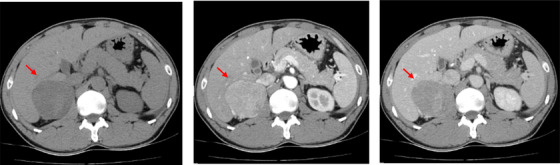
Adrenal CT scan. Left panel: routine scan; Middle panel: arterial phase; Right panel: venous and delayed phase

Twenty‐four hour ambulatory systolic/diastolic blood pressure was on average 210/125 mmHg. Abdominal ultrasound showed that the size of left kidney was 107 mm*46 mm, and right kidney 93 mm*49 mm. Doppler showed no abnormalities in renal artery blood flow. The glomerular filtration rate (GFR) measured with single photon emission computed tomography (SPECT) was 44.4 and 38.5 mL/min for the left and right kidneys, respectively. CT scan showed a mild‐density rounded occupation on the right adrenal gland in a size of 63 mm*60 mm with a clear margin. After enhancement, the arterial phase was obviously strengthened, and the venous phase was somewhat reduced. The shape of the left adrenal gland was normal, and no abnormal density was observed, indicating a unilateral right side adrenal mass (Figure 1).

After admission, 9 g of oral potassium supplementation daily was given, and sufficient dosages of a non‐dihydropyridine calcium antagonist and an α‐blocker were used to control blood pressure. Blood pressure remained elevated. Amlodipine was added. Diastolic blood pressure was still in the range from 110–120 mmHg.

To exclude adrenal malignancy, the function of zona fasciculate was reviewed, increased UFC (533.25 μg/24 h, reference range: 58–403 μg/24 h) was found, and circadian rhythm of serum cortisol disappeared. The 2 mg‐dexamethasone suppression test was positive, and adrenocorticotropic hormone (ACTH) decreased to 4.77 pg/mL (Table 1). As the patient was young (< 35 years of age) with spontaneous hypokalemia, marked aldosterone excess, and unilateral adrenal lesions with radiological features consistent with a cortical adenoma, adrenal venous sampling (AVS) was not performed.^8^

**TABLE 1:** 2 mg‐dexamethasone suppression test

**Phase**	**Serum cortisol (μg/dL)**	**ACTH (pg/mL)**	**24‐h urine free cortisol (μg/24h)**
**Basis**	11.57	4.77 ↓	533.3 ↑
**1^st^ day after medication**	10.58	5.22 ↓	693.0 ↑
2^st^ day after medication	11.16	6.24 ↓	624.0 ↑

ACTH, adrenocorticotropic hormone.

The diagnosis was secondary hypertension; primary aldosteronism and subclinical Cushing's syndrome, with a unilateral adrenal mass (aldosterone and cortisol co‐secreting adenoma, right side).

## 3 METHODS

**FIGURE 2:**
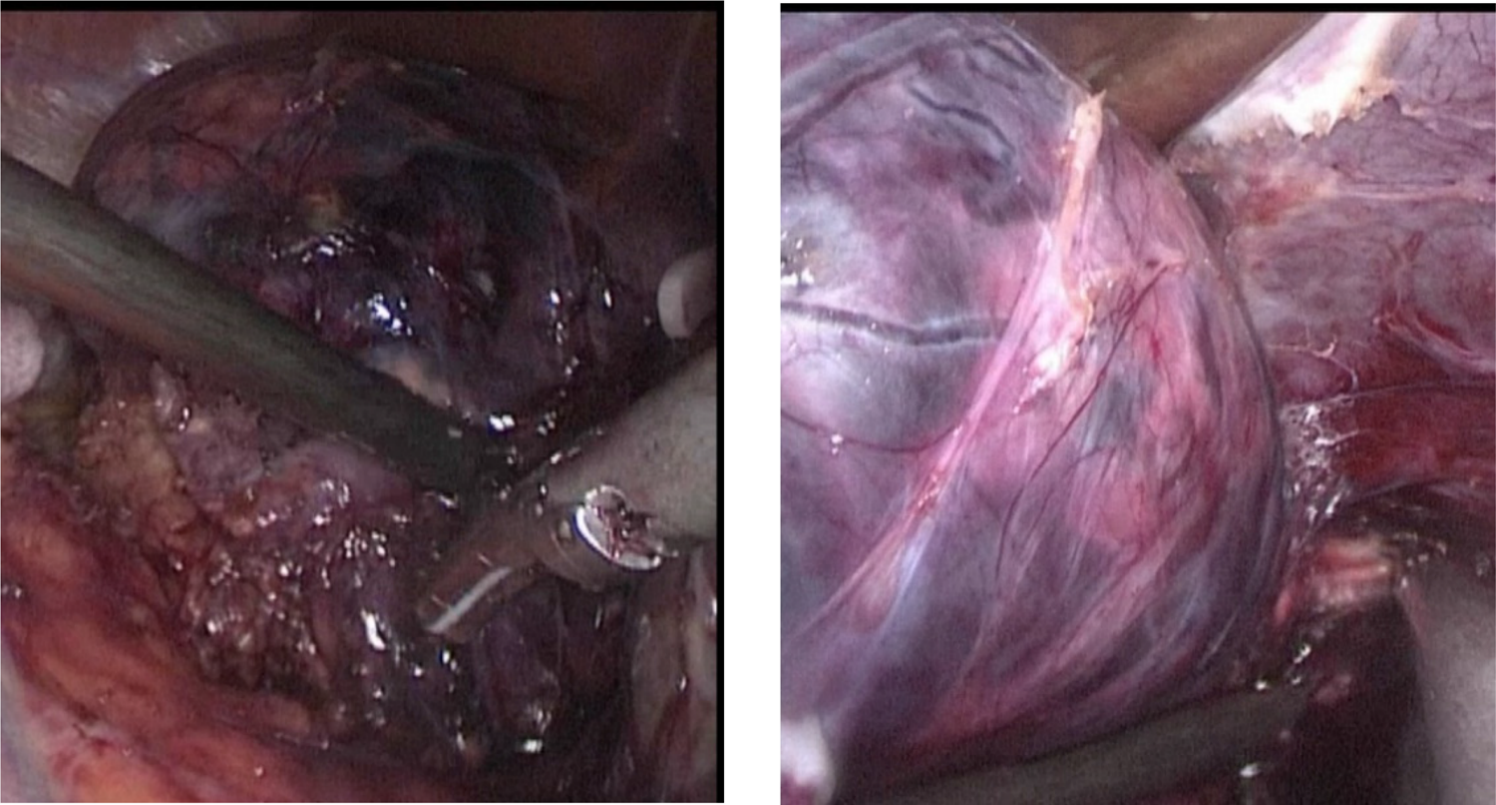
Laparoscopic unilateral adrenalectomy (right side)

The treatment choice was surgery. To prepare for the surgery, the patient was then given spironolactone 60 mg three times daily in combination with the previously prescribed antihypertensive drugs to control blood pressure and oral potassium supplement to correct hypokalemia. After 2 weeks of pre‐operation preparation, the patient underwent unilateral adrenalectomy (Figure 2). One hundred milligram hydrocortisone was used during surgery, and followed oral administration of low dose glucocorticoid after surgery for two days.

## 4 RESULTS

**FIGURE 3:**
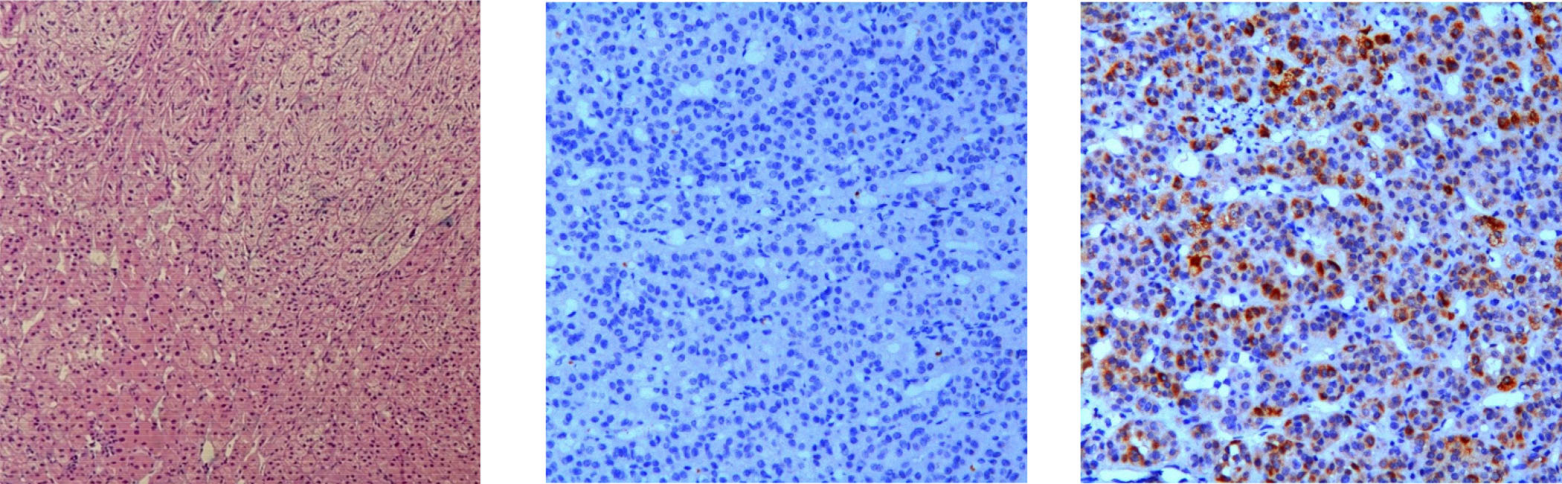
Histopathological findings. Left panel: hematoxylin‐eosin staining; Middle panel: CYP11B1 Immunostaining (‐); Right panel: CYP11B2 immunostaining (+)

Hematoxylin–eosin staining showed fasciculate (ZF) like cells, which were large, lipid laden clear, with round to oval vesicular nuclei and zona glomerulosa (ZG) like cells, characterized as small, compact cells, with high nuclear/cytoplasmic ratio and moderate amount of lipid. Immunohistochemistry showed CYP11B1 (11β‐hydroxylase) negative and CYP11B2 (aldosterone synthase) positive (Figure 3).

After 1, 3, and 4 months of surgery, candesartan and amlodipine were prescribed, and systolic/diastolic blood pressure was around 130/90 mmHg. Plasma aldosterone, PRA, serum cortisol and ACTH returned to the normal range (Table 2). After 1 year of surgery, no antihypertensive medication or cortisol was prescribed, and blood pressure was normal.

**TABLE 2:** Pre‐ and post‐operative serum level of hormone

		After surgery	
Variable	Preoperative	1 month	3 months
Serum potassium concentration (mmol/L)	2.42	4.54	3.93
Plasma aldosterone concentration (pg/mL)	715.72	50.40	46.19
Plasma renin activity (ng/ml/h)	0.04	0.04	1.46
Serum cortisol concentration (μg/dL)	11.57	10.24	–
Serum ACTH concentration (pg/mL)	4.77	41.31	–
24‐h urine free cortisol excretion (μg/24h)	533.25	–	–

ACTH, adrenocorticotropic hormone.

## 5 DISCUSSION

Because only one case has been found among approximately 177 diagnosed patients with primary aldosteronism in a ward of our department, the coexistence of subclinical Cushing syndrome can still be considered rare. However, identification of subclinical Cushing's syndrome in primary aldosteronism might not only help for correct lateralization but also for the postoperative management, such as glucocorticoids supplementation for the prevention of adrenocortical insufficiency or even adrenal crisis.^9^

In 1977, Hogan and associates first reported primary aldosteronism with subclinical Cushing's syndrome, which was initially considered to be a rare disease.^1^ Recent studies showed that the prevalence of subclinical Cushing's syndrome could be higher up to 21% (8/38) in patients with aldosterone‐producing adenoma^10^ and 12.1% in adrenal incidentaloma.^11^ The size of the aldosterone‐producing adenoma matters for the identification of subclinical Cushing's syndrome.^9^, ^10^ Such patients all had large (2.0‐2.5 cm in diameter) adenomas, minimally 1.1 cm.^12^ Adenomas are usually in a single neoplasm that secrets both aldosterone and cortisol, but can also be in two different neoplasms, either ipsilateral or contralateral, that secret aldosterone and cortisol, respectively.^9^ If the diameter of the aldosterone‐producing adenoma is 2.0‐2.5 cm, subclinical Cushing's syndrome has to be considered. In addition to serum and 24‐h urinary free cortisol, a low dose dexamethasone suppression test is required for the diagnosis of subclinical Cushing's syndrome.^2^, ^9^

In the present case, AVS was not performed according to endocrine society clinical practice guideline for management of primary aldosteronism that younger patients (< 35 years) with spontaneous hypokalemia, marked aldosterone excess, and unilateral adrenal lesions with radiological features consistent with a cortical adenoma on adrenal CT scan may not need AVS before unilateral adrenalectomy.^8^ However, it is noteworthy that whether the recommendation can be implemented in primary aldosteronism combined with subclinical Cushing's syndrome. Moreover, the combined presence of aldosterone‐producing adenoma with subclinical Cushing's syndrome may mislead the results of AVS. In the presence of adenoma that secrets both aldosterone and cortisol, cortisol adjustment for aldosterone would not be useful for lateralization. Metanephrine instead of cortisol‐adjustment for aldosterone might be required.^13^ In the case of cortisol‐secreting neoplasm with contralateral aldosterone‐producing (micro‐)adenoma, although lateralization is still possible and correct, unilateral adrenalectomy for the removal of aldosteronoma might not be clinically useful for blood pressure control.^10^ Cortisol hypersecretion of the remaining adrenal gland may still cause high blood pressure. Surgery therefore should be cautiously considered. The results of recent studies suggested that super‐selective adrenal venous sampling (ssAVS) may be suitable for the subtype diagnosis of primary aldosteronism with subclinical Cushing's syndrome.^14^, ^15^

ssAVS was developed using a micro‐catheter, which collects blood samples from adrenal tributary veins. Cortisol‐adjustment was not required for samples contain a limited amount of systemic venous blood. Absolute value of plasma aldosterone concentrations and plasma cortisol concentrations were utilized when analyzed, thus can avoid misjudgment.^15^

The histopathological characterization of aldosterone and cortisol co‐secreting adenoma could show ZF and ZG like cells, respectively, while CYP11B1 and CYP11B2 immunostaining demonstrated heterogeneity. It may be expressed as CYP11B1 and CYP11B2 both positive, or low or lack expression of CYP11B1 or CYP11B2. The interpretation of the latter was that aldosterone and cortisol cosecreting adenoma was composed of numerous aldosterone‐secreting and cortisol‐secreting cells, and even if the production per cell was low, the overall amount was still large, thus negative or low expression of CYP11B1 or CYP11B2 may occur.^16^

In conclusion, in patients with aldosterone‐producing adenoma, the function of zona fasciculate might have to be evaluated. The presence of subclinical Cushing's syndrome may not only influence lateralization but also post‐surgery management. A low serum ACTH concentration might behave as an indicator for subclinical Cushing's syndrome.
